# Retraction: Effects of mandibular extension on pial arteriolar diameter changes in glucocorticoid-induced hypertensive rats

**DOI:** 10.3389/fphys.2026.2026350

**Published:** 2026-10-19

**Authors:** Frontiers Editorial Office

Following publication, concerns were raised regarding the integrity of the images in the published figures. The authors failed to provide a satisfactory explanation during the investigation, which was conducted in accordance with Frontiers’ policies. As a result, the data and conclusions of the article have been deemed unreliable and the article has been retracted.

The retraction was approved by the Chief Editors of Frontiers in Physiology and the Chief Executive Editor at Frontiers. The authors were informed of the retraction and had an opportunity to respond. This communication has been recorded by the publisher.

